# Antimicrobial resistance profiling and burden of resistance genes in zoonotic *Salmonella* isolated from broiler chicken

**DOI:** 10.1002/vms3.648

**Published:** 2021-10-02

**Authors:** Tridip Das, Eaftekhar Ahmed Rana, Avijit Dutta, Md. Bayazid Bostami, Mizanur Rahman, Probir Deb, Chandan Nath, Himel Barua, Paritosh Kumar Biswas

**Affiliations:** ^1^ Department of Microbiology and Veterinary Public Health Chattogram Veterinary and Animal Sciences University Khulshi Chattogram Bangladesh; ^2^ Poultry Research and Training Centre Chattogram Veterinary and Animal Sciences University Khulshi Chattogram Bangladesh; ^3^ Teaching and Training Pet Hospital and Research Centre Chattogram Veterinary and Animal Sciences University Bangladesh

**Keywords:** antimicrobial resistance, chicken, resistance genes, *Salmonella*

## Abstract

**Background:**

*Salmonella* is frequently found in poultry of which only motile serovars have zoonotic significance due to their potential to induce human gastrointestinal infections. Antimicrobial resistance, being a public health concern, the emergence of multidrug‐resistant (MDR) *Salmonella* serotypes affecting food chain has greater impact worldwide.

**Aim:**

Information on circulation of zoonotic *Salmonella* strains in commercial poultry farm level is limited in many parts of the world. This cross‐sectional study was aimed to investigate the zoonotic *Salmonella* strains circulating in the broiler farm environment with their detailed antimicrobial resistance profiling.

**Methods:**

Pooled faecal samples were collected randomly from commercial broiler farms of Chattogram district, Bangladesh. Standard bacteriological procedure was followed to isolate *Salmonella*, and identification was confirmed by genus specific polymerase chain reaction (PCR). After phenotypic characterisation of resistance profile against eight antimicrobials by disc diffusion technique, all strains were screened by PCR for some selected resistance genes_._

**Results:**

Out of the 350 samples, *Salmonella* was isolated and identified from 86 samples. In antimicrobial sensitivity testing, more than 98.8% isolates showed resistance to ampicillin and 94.2% to tetracycline followed by enrofloxacin (56%) and ciprofloxacin (50%). Notably, 94% isolates were found to be MDR. The results of PCR assays revealed that 81.4% of the isolates were carrying the *tetA* gene, 19.8% the *tetB* and 10.47% the *tetC* gene. The prevalence of the isolates bearing the *bla*_TEM_, *bla*_CTX‐M_ and *Sul*‐I gene were 95.4%, 7.0 % and 37.2 %, respectively.

**Conclusion:**

There is a great risk to secure healthy poultry products due to the circulation of these MDR zoonotic *Salmonella*

## Graphical abstract

This cross‐sectional study was aimed to investigate the zoonotic *Salmonella* strains circulating in the broiler farm environment with their detailed antimicrobial resistance profiling. Out of the 350 samples, prevalence of *Salmonella* was 24% and multidrug‐resistant (MDR) *Salmonella* was 94%. There is a great risk to secure healthy poultry products due to the circulation *tetA*, *tetB*, *tetC*, *bla*_TEM_, *bla*_CTX‐M_ and *Sul*‐I antimicrobial resistance genes in these MDR zoonotic *Salmonella*.

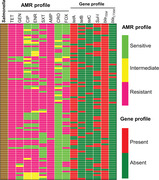

## 1 INTRODUCTION

*Salmonella* is one of the major food‐borne pathogens throughout the world. *Salmonellae* are gram negative, non‐spore forming, non‐capsulated, aerobic and facultative anaerobic rod and classified under the family *Enterobacteriaceae* (OIE, 2006). They include a large group of serologically and biochemically related bacilli and are motile by means of peritrichous flagella with the exception of *Salmonella* Pullorum and *Salmonella* Gallinarum (Grimont et al., 2000). More than 2600 serovars exist based on 67 ‘O’ and the 117 ‘H’ antigens (for motile species) recognised so far (Grimont & Weill, 2007; Popoff & Le Minor, 2001). According to the level of host association, the three main groups of *Salmonella* serovars are host restricted, host adapted and generalised (Uzzau et al., 2000). All motile serovars of poultry origin are thought to be zoonotic and most of them pose a threat to public health. Among different zoonotically important *Salmonella* serovars, the most frequently worldwide reported serovars are *S*. Typhimurium and *S*. Enteritidis (Chiu et al., 2010). Most often, human infection has been attributed to consumption of poultry products such as eggs and meat contaminated with these zoonotic strains.

A number of clinical cases caused by *Salmonella* both in human and animal have been reported annually all over the world (Hoelzer et al., 2011; Meakins et al., 2008). This is why it is a global issue not only for acquiring resistance of its many strains to several critically important antimicrobial agents but also for their potential transmission to humans. In an investigation in the last decade, 53.9% of non‐typhoidal *Salmonella* isolates from chickens were resistant to at least one antimicrobial agent (Food and Drug Administration, 2010). Since, antimicrobial uses are not well‐monitored in most developing countries, their misuse and overuse by poultry farmers to protect their birds from infections are commonly reported in such poorly regulated settings. This irrational use of antimicrobials might lead to the emergence of antimicrobial resistance in bacterial pathogens such as zoonotic *Salmonella*. Through the trades of poultry and poultry products and human movements, this drug‐resistant *Salmonella* can spread beyond the national borders. Local emergence of a multidrug‐resistant (MDR) *Salmonella* strain in poultry has therefore a far‐reaching impact apart from the source of origin and circulation.

All the poultry production systems, as classified by Food and Agriculture Organization of the United Nations, exist in Bangladesh; however, small‐scale commercial production predominates (FAO, 2005). In such farming, birds might be more vulnerable to become exposed to *Salmonella* (Parvej et al., 2016). To control *Salmonella* in poultry farms of an area, it is important to know its magnitude of the infection in this population. With the exception of two previous studies in Bangladesh (Barua et al., 2012, 2013), there is very little information available on the distribution of zoonotic *Salmonella* strains in broiler chicken. This study was aimed to fill the gap in knowledge on baseline information as mentioned above through investigating the zoonotic *Salmonella* circulating in the broiler farm environment in Bangladesh along with unveiling the phenotypic and genotypic patterns of the strains acquiring some selected antimicrobial resistant determinants.

## 2 MATERIALS AND METHODS

### 2.1 **Study** population and sampling

This cross‐sectional study was conducted in Chattogram (previously Chittagong) district (administrative unit), the second largest of all the 64 districts in Bangladesh. Samples were collected from 350 randomly selected broiler farms during the period of July 2018 to June 2019. A single pooled faecal sample was collected from each farm. Each sample consisted of five naturally pooled faecal samples collected from five different locations from the same floor of a farm. Each pooled sample consisted of ∼25 cross‐sectional pinches of faeces mixed with litter for obtaining a total weight of approximately 200 gm. After collection of such a pooled sample, it was placed separately into a sterile plastic zipper bag and brought to the laboratory of the Department of Microbiology and Veterinary Public Health, Chattogram Veterinary and Animal Sciences University, Bangladesh.

### 2.2 **Isolation** of ***Salmonella***

For isolation of *Salmonella*, standard bacteriological procedures were followed. Briefly, after the pre‐enrichment of the pooled samples in buffered peptone water (Oxoid Ltd.), it was inoculated on Modified Semi‐solid Rappaport‐Vassiliadis (MSRV) medium (Himedia Ltd.) supplemented with novobiocin (HiMedia Ltd.) and incubated at 41.5°C for 24–36 h. Later, inoculum from any swarming growth observed on the MSRV plates was transferred to brilliant‐green agar (Oxoid Ltd.) and incubated overnight at 37°C to obtain isolated colonies. Suspected *Salmonella* colonies were cultured onto blood agar and stored at −80°C for further examination.

### 2.3 **Identification** of ***Salmonella*** by polymerase chain reaction (PCR)

Genomic DNA was extracted by the crude boiling method (Dashti et al., 2009). Later, suspected isolates were confirmed by conventional PCR assay using *Salmonella* genus‐specific primers ST‐11 (5ʹ ‐AGCCAACCATTGCTAAATTGGCGCA‐3ʹ) and ST‐15 (5ʹ‐TGGTAGAAATTCCCAGCGGGTACTG‐3ʹ; Gouws et al., 1998). Amplification was done with 25‐μl total reaction volume for characteristic 429‐bp PCR product by maintaining the initial denaturation at 94°C for 2 min followed by 35 cycles at 95°C for 30 s, 60°C for 30 s and 72°C for 30 s and then one final step with 10 min of extension at 72°C (Gouws et al., 1998). *Escherichia coli* ATCC 25922 and a *Salmonella* Kentucky in‐house strain were used as negative and positive control, respectively.

### 2.4 **Antimicrobial** susceptibility testing (AST)

AST of *Salmonella* isolates was conducted by disc diffusion method according to Clinical and Laboratory Standards Institute (CLSI) guidelines (CLSI, 2018). A total of eight antimicrobials from six different groups were included for AST at the indicated concentrations: ampicillin (10 μg), cefoxitin (30 μg), ceftriaxone (30 μg), ciprofloxacin (5 μg), enrofloxacin (5 μg), gentamicin (10 μg), sulfamethoxazole/trimethoprim (25 μg) and tetracycline (30 μg). The results of the AST were interpreted as resistant, intermediate and sensitive according to standards provided by CLSI (CLSI, 2018). If any isolate displayed resistance to more than two different classes of antimicrobials, it was defined as ‘MDR’ (Weill et al., 2006).

### 2.5 **Detection** of antimicrobial resistance genes

All *Salmonella* isolates were tested for the presence of the *tetA*, *tetB* and *tetC*, s*ul‐*I, *bla*_TEM_ and *bla*_CTX‐M_ genes by PCR assay using the specific sets of primers as described earlier (Table 1). The PCR conditions for all the resistance genes were described in Table 1. *Escherichia coli* ATCC 25922 was used as the negative control, and three *Salmonella* in‐house strains carrying the tested genes were used as the positive controls.

**TABLE 1:** Primer sequences used in polymerase chain reaction (PCR) to detect antimicrobial resistance genes

**Gene**	**Primer name**	**Primer sequence (5΄‐ 3΄)**	**Amplicon size (bp)**	**PCR condition**	**Reference**
*tetA*	*tetA*‐F *tetA*‐R	GGCGGTCTTCTTCATCATGC CGGCAGGCAGAGCAAGTAGA	502	Initial denaturation at 95°C for 4 min, 35 cycles of denaturation at 95°C for 1 min, annealing at 64°C for 1 min, extension at 72°C for 1 min and final extension at 72°C for 7 min	(Lanz et al., 2003)
*tetB*	*tetB*‐F *tetB*‐R	CATTAATAGGCGCATCGCTG TGAAGGTCATCGATAGCAGG	930		(Lanz et al., 2003)
*tetC*	*tetC*‐F *tetC*‐R	GCTGTAGGCATAGGCTTGGT GCCGGAAGCGAGAAGAATCA	888		(Lanz et al., 2003)
*Sul*‐I	*Sul*I‐F *Sul*I‐ R	CGG CGT GGG CTA CCT GAA CG GCC GAT CGC GTG AAG TTC CG	779	Initial denaturation at 95°C for 5 min, 35 cycles of denaturation at 95°C for 1 min, annealing at 68°C for 1 min, extension at 72°C for 1 min and final extension at 72°C for 10 min	(Lanz et al., 2003)
*bla*_TEM_	*bla*_TEM_ F *bla*_TEM_ R	GCGGAACCCCTATTTG TCTAAAGTATATATGAGTAAACTTGGTCTGAC	964	Initial denaturation at 94°C for 3 min, 25 cycles of denaturation at 94°C for 1 min, annealing at 50°C for 1 min, extension at 72°C for 1 min and final extension at 72°C for 10 min	(Hasman et al., 2005)
*bla*_CTX‐M_	CTXMF CTXMR	ACGCTGTTGTTAGGAAGTG TTGAGGCTGGGTGAAGT	857	Initial denaturation phase of 94°C for 3 min and then 36 cycles of 94°C for 1 min, 58°C for 30 s, 72°C for 1 min and 72°C for 10 min	(Feizabadi et al., 2010)

### 2.6 **Statistical** analysis

All data were entered into a spreadsheet of Microsoft Excel 2016 and transferred to R 3.5.1 (R Core Team, 2016) for data summary and analysis. The geographical coordinates of the farms were recorded while collecting samples from the farms. A spot map of *Salmonella* positive farms was created with QGIS 2.18.13 (Westra, 2014). The heatmap showing the distribution of antimicrobial resistance phenotype and genotype of *Salmonella* isolates was prepared by using GraphPad Prism 7 (Mitteer et al., 2018).

## 3 RESULTS

### 3.1 **Prevalence** of zoonotic ***Salmonella***

**FIGURE 1:**
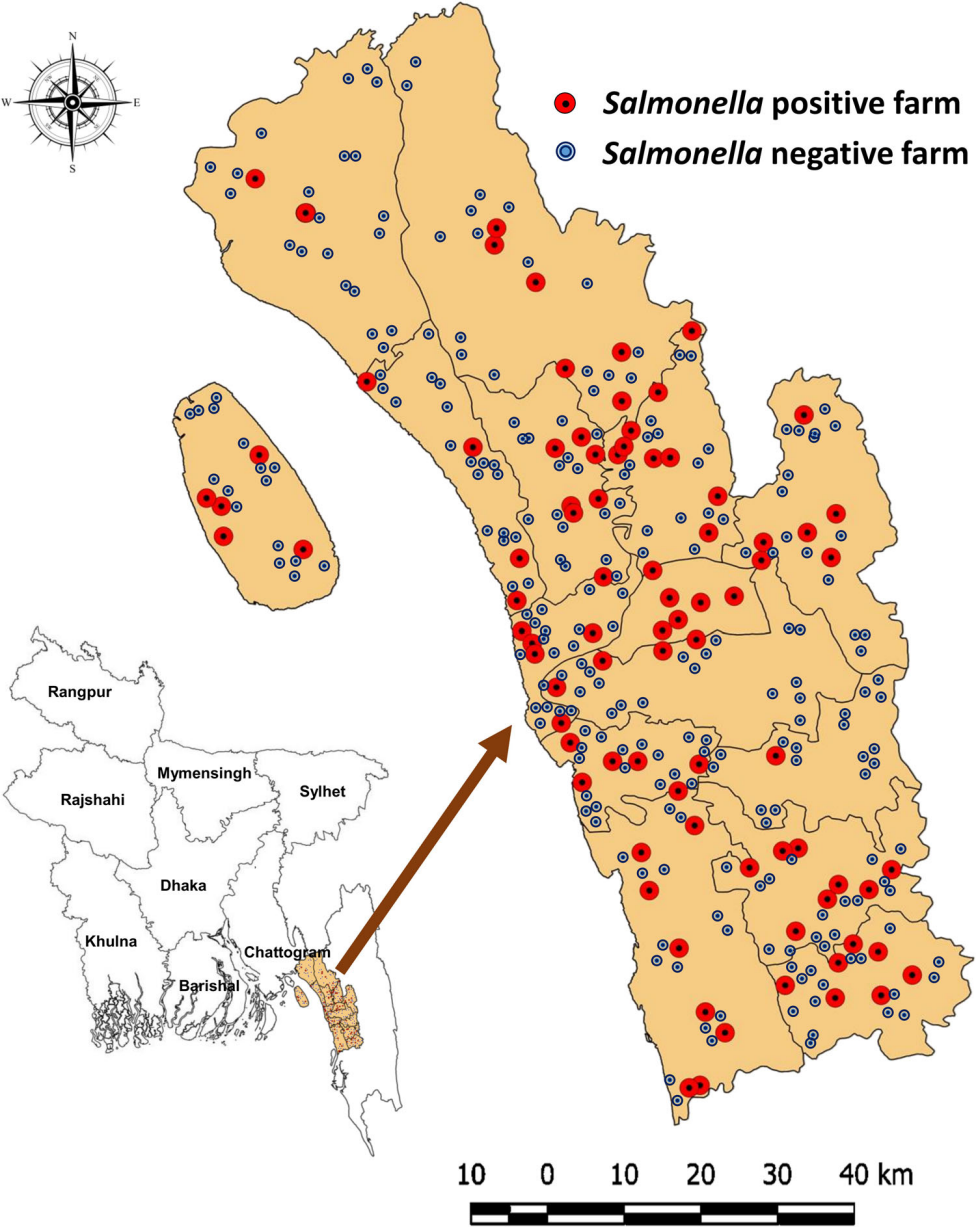
Geographical distribution of *Salmonella* positive and negative broiler farms, where each red‐ or blue coloured circle indicates single broiler farm

A total of 350 samples were collected from the study area. Among them 86 (24.57%; 95% confidence interval (CI), 20.3%–29.4%) were positive for *Salmonella*. All *Salmonella‐*positive farms were of wider geographical locations as portrayed in Figure 1.

### 3.2 **Antibiogram** profiles of ***Salmonella*** isolates

**FIGURE 2:**
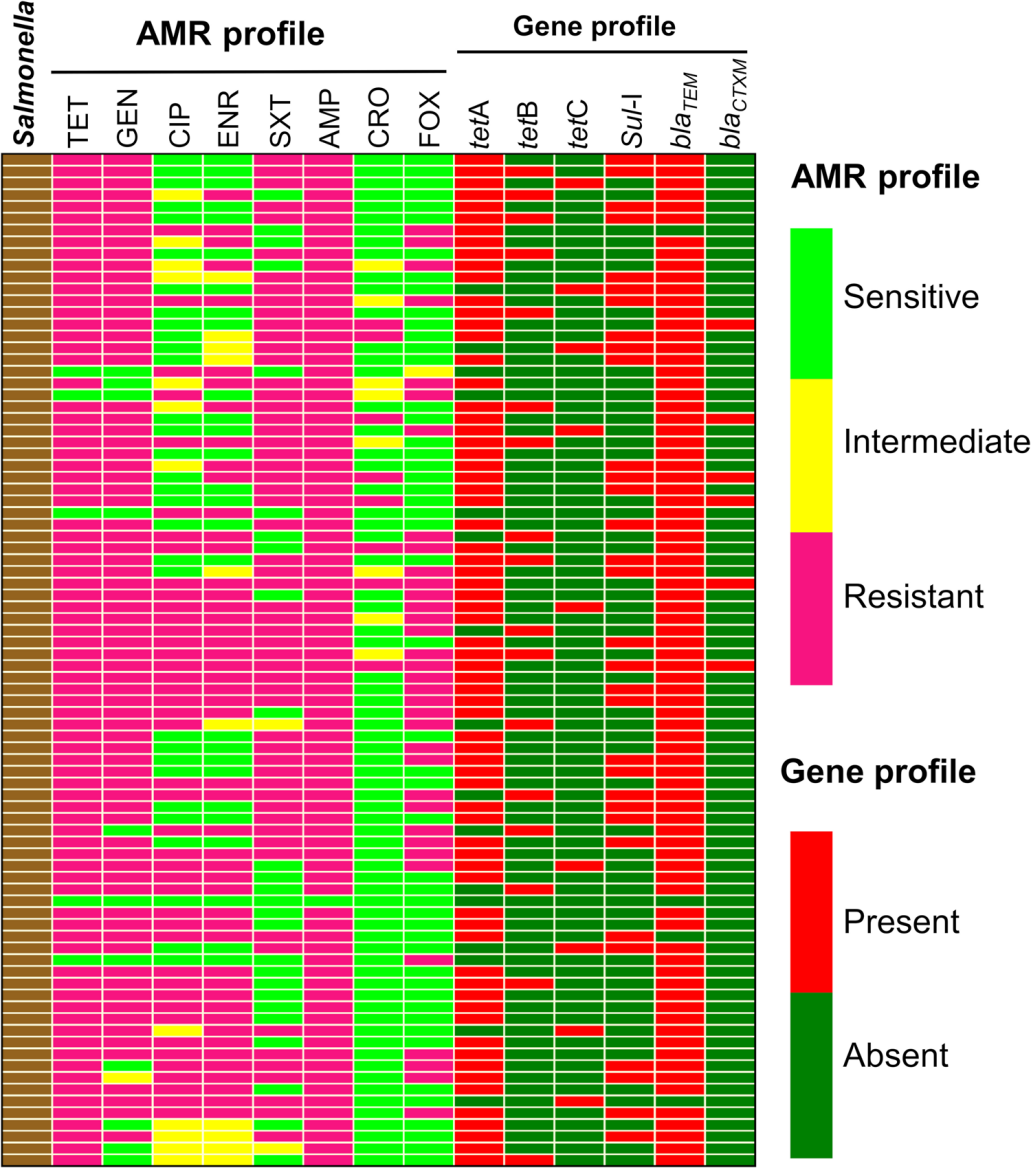
Distribution of antimicrobial resistance phenotype and genotype of *Salmonella* isolates, where TET = tetracycline, GEN = gentamicin, CIP = ciprofloxacin, ENR = enrofloxacin, SXT = sulfamethoxazole/trimethoprim, AMP = ampicillin, CRO = ceftriaxone, FOX = cefoxitin

**FIGURE 3:**
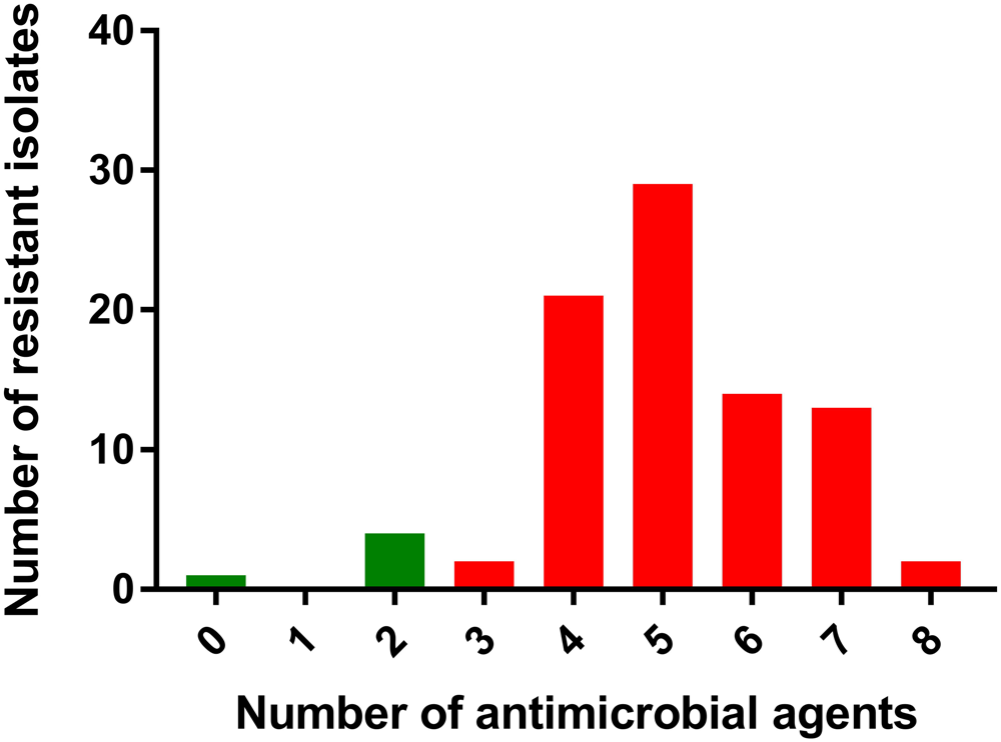
Multidrug‐resistance profile of all *Salmonella* isolates [*n* = 86], where red bars indicate multidrug‐resistant (MDR) isolates and green bars indicate other than MDR isolates

The results of AST of all the *Salmonella* isolates are shown in Table 2. The results showed that more than 94% isolates were resistant to ampicillin and tetracycline, but only 9.3% and 40.7% to ceftriaxone and cefoxitin, respectively. Individual antibiogram profiles of all the isolates are displayed in Figure 2. The prevalence of MDR *Salmonella* isolates was 94%, two‐third of them showing resistance against 4–6 antimicrobials tested (Figure 3).

**TABLE 2:** Antimicrobial resistance pattern of *Salmonella* isolates [*n* = 86] obtained from broiler chicken

**Antimicrobial agents**	**Sensitive (%)**	**Intermediate (%)**	**Resistant (%)**
Ampicillin	1 (1.16)	0 (0)	85 (98.84)
Cefoxitin	50 (58.14)	1 (1.16)	35 (40.70)
Ceftriaxone	70 (81.40)	8 (9.30)	8 (9.30)
Ciprofloxacin	31 (36.05)	12 (13.95)	43 (50.00)
Enrofloxacin	27 (31.40)	10 (11.63)	49 (56.98)
Gentamicin	11 (12.79)	1 (1.16)	74 (86.05)
Sulfamethoxazole/trimethoprim	26 (30.23)	2 (2.33)	58 (67.44)
Tetracycline	5 (5.81)	0 (0)	81 (94.19)

### 3.3 **Distribution** of antimicrobial resistance genes

Among the isolates tested 81.4% (95% CI, 71.8%–88.3%) carried the *tetA* gene followed by 19.8% (95% CI, 12.63%–29.49%) the *tetB* gene and 10.5% (95% CI, 5.4%–18.9%) the *tetC* gene. All the isolates harbouring one of the three tet genes were phenotypically resistant to tetracycline (Figure 2). The occurrences of tetracycline, sulfamethoxazole/trimethoprim, ampicillin and ceftriaxone resistance genes among *Salmonella* isolates are shown in Table 3. Out of the 86 isolates, 37.2% (95% CI, 27.7%–47.9%) were found positive for the presence of the *Sul‐*I gene. The *bla*_TEM_ gene was detected in 95.4%% (95% CI, 88.3%–98.5%) isolates, whereas the *bla*_CTX‐M_ gene in 7.0 % (95% CI, 3.0%–14.7%).

**TABLE 3:** Occurrence of antimicrobial resistance genes among *Salmonella* isolates [*n* = 86] from broiler chicken

**Antimicrobials**	**Resistance genes**	**Number of resistant isolates**	**Prevalence (95% confidence interval)**
Tetracycline	*tetA*	70	81.40 (71.79–88.32)
	*tetB*	17	19.77 (12.63–29.49)
	*tetC*	9	10.47 (5.40–18.91)
Sulfamethoxazole/trimethoprim	*Sul*‐I	32	37.21 (27.73–47.78)
Ampicillin	*bla*_TEM_	82	95.35 (88.28–98.54)
Ceftriaxone	*bla*_CTX‐M_	6	6.98 (2.95–14.68)

## 4 DISCUSSION

The results of the present study revealed that zoonotic *Salmonella* strains are circulating in commercial broiler poultry farms in Bangladesh. The overall prevalence of zoonotic *Salmonella* was 24.6%. Although this prevalence was quite similar to the result obtained from another study carried out in the same geographical location (Al Mamun et al., 2017), the previous study failed to characterise whether the isolates obtained were motile, which are zoonotic or non‐motile and are poultry host‐specific. In contrast, few studies in broiler and layer poultry farms of the same region showed a bit lower prevalence (Barua et al., 2012; Barua et al., 2013; Hossain et al., 2015). The prevalence estimates of *Salmonella* in broiler farms were reportedly variable from as low as 10% to as high as 37% or even higher irrespective of geographical variation (Asif et al., 2017; Dione et al., 2009; Elgroud et al., 2009; Salles et al., 2008; Samanta et al., 2014; Snow et al., 2008).

In this study, all the isolates displayed a high level of resistance against routinely used antimicrobials. Strong selective pressure by exposure to regularly used antibiotics could be one of the main causes for the emergence of such antibiotic‐resistant *Salmonella* strains (Wright, 2007). A total of 81 (94%) isolates identified in this study were classified as MDR. The possible reason for the development of resistance might be linked to the excessive and irrational use of antibiotics with improper dosages and schedules in commercial poultry farming. Plasmid‐mediated horizontal transfer of antimicrobial resistance gene(s) may play important role in seeing such a high rate of drug resistance among the isolates (Carattoli, 2003). The antimicrobial against which isolates showed the highest sensitivity (81.4%) was ceftriaxone. This is a reserved antimicrobial for human clinical treatment, and it should be a matter of investigation why 18% of *Salmonella* isolates obtained from the study were resistant against it.

It was apparent that resistance to classical antibiotics and detection of their respective resistance gene(s) in microbial populations were in a high proportion. Among all the resistance genes investigated, *tet* genes occurred most frequently in our study. The prevalence of *tetA*, *tetB* and *tetC* among the isolates was 81.4%, 19.8% and 10.5%, respectively, where *tetA* was found as the most prevalent tetracycline resistance gene, an agreement with the findings of some previous studies (Adesiji et al., 2014; McDermott et al., 2016). Around 37.21% isolates harboured the *sul*‐I gene responsible for sulfonamides resistance. The presence of *bla*_TEM_ gene in the *Salmonella* isolates was 95.4%, which was the highest among all the resistance genes studied, and the results revealed that almost all ampicillin‐resistant isolates possessed the *bla*_TEM_ gene (Adesiji et al., 2014; Olesen et al., 2004). On the other hand, the ceftriaxone resistance gene, namely, *bla*_CTX‐M_ was circulating in a low frequency (6.98%) among the isolates isolated, and the reason behind such low prevalence of the gene might be linked to its low or no use in poultry farming in Bangladesh.

The overall characteristics of the farms studied (e.g., flock size, rearing system, management practices, etc.) were mostly similar across the study area and the period of the study (data not shown). Most of the farms investigated had minimum biosecurity facilities and practices with easy access to people, wild birds, animals and rodents. Studies showed that wild birds and rodents play a pivotal role in the transmission and spillover of *Salmonella* within and in between farms as they act as the carrier of *Salmonella* (Bouzidi et al., 2012; Kinde et al., 2005). Simultaneously, the presence of any non‐host specific motile *Salmonella* in poultry is a public health concern in relation to food safety. The spread of this zoonotic pathogen from infected or carrier birds to healthy chickens of farms as well as in retail outlets at the local live bird markets pose a potential risk to public health. Therefore, control measures need to be executed to limit the spread of zoonotic *Salmonella*.

## 5 CONCLUSION

In conclusion, this study provides critical baseline information and scientific evidence on the circulation of MDR zoonotic *Salmonella* strains in poultry in Bangladesh. The possibility of transmission of them to humans via the food chain is a potential threat to public health locally and beyond. There is also a great risk to secure healthy poultry products due to the circulation of these MDR strains of zoonotic *Salmonella*. Therefore, further intervention studies are recommended to explore the risk factors associated with zoonotic *Salmonella* in poultry in order to mitigate them as part of effecting control for zoonotic *Salmonella* from entering poultry to the human food chain.

## ETHICS STATEMENT

This study was approved by the ethical committee of Chattogram Veterinary and Animal Sciences University, Bangladesh with retrospective effect from the date of its commencement. The memo no. is CVASU/Dir(R&E) EC/2019/39(2/6/6).

## AUTHOR CONTRIBUTION

Tridip Das designed and carried out this project. Tridip Das, Eaftekhar Ahmed Rana, Avijit Dutta, Mizanur Rahman, Md. Bayazid Bostami, Probir Deb and Chandan Nath contributed in sample collection, data collection, lab works and manuscript preparation. Tridip Das analysed data prepared first draft. Himel Barua and Paritosh Kumar Biswas supervised the study and checked the writing of this article.

## CONFLICT OF INTEREST

The authors have no potential conflict of interest.

### PEER REVIEW

The peer review history for this article is available at https://publons.com/publon/10.1002/vms3.648

## Data Availability

The data supporting the findings of this study are available within the article.
